# Direct visualization-guided endoscopic management of complex colonic diverticular disease: a proof-of-concept case series

**DOI:** 10.3389/fmed.2026.1929061

**Published:** 2026-09-03

**Authors:** Tianjie Han, Junshan Li

**Affiliations:** 1 Departments of Hematology, Shandong Provincial Third Hospital, Shandong University, Jinan, Shandong, China; 2 Department of Gastroenterology, Shandong Provincial Third Hospital, Shandong University, Jinan, Shandong, China

**Keywords:** colonic diverticular disease, direct visualization, diverticular bleeding, endoscopy, EyeMax system, inflammatory diverticular disease

## Abstract

**Background:**

Complex colonic diverticular disease, including diverticular bleeding, inflammatory diverticular disease, presents substantial therapeutic challenges for conventional colonoscopy because of limited intradiverticular visualization, difficult access, and restricted maneuverability. Failure to precisely localize the culprit lesion or inflammatory nidus may lead to recurrent symptoms, delayed treatment, or surgical intervention. The EyeMax direct visualization system, originally designed for pancreatobiliary interventions, may provide a novel strategy for targeted intradiverticular diagnosis and therapy.

**Aim:**

To evaluate the feasibility, safety, and potential clinical applications of the EyeMax direct visualization system in the endoscopic management of complex colonic diverticular disease.

**Methods:**

We conducted a retrospective proof-of-concept case series of patients with complex colonic diverticular disease who underwent EyeMax-assisted endoscopic intervention at a tertiary hospital between January 2024 and July 2026. Included patients had diverticular bleeding, diverticulitis with localized inflammation or purulent cavity, or diverticular fecalith incarceration. Primary outcome was technical success, defined as successful intradiverticular access and completion of intended intervention under direct visualization. Secondary outcomes included clinical success, adverse events, recurrence, and need for surgery.

**Results:**

Seven patients were included, comprising diverticular bleeding (*n* = 2), inflammatory diverticular disease(*n* = 5). EyeMax-assisted intervention achieved a technical success rate of 100% (7/7). Clinical success was achieved in 85.7% (6/7) of patients. Among the five patients with inflammatory diverticular disease, direct intradiverticular exploration identified purulent cavities and inflammatory debris in all cases, with impacted fecaliths detected in three patients. Four patients improved following direct visualization-guided lavage with or without fecalith extraction, whereas one patient with severe diverticulitis complicated by abscess formation required surgical intervention. No procedure-related perforation, delayed bleeding, infection, or mortality occurred. During follow-up ranging from 1 month to 2 years, no recurrent bleeding or inflammatory events were observed.

**Conclusion:**

In this preliminary assessment, the EyeMax system demonstrates feasibility for managing complex colonic diverticular disease, with potential utility in diverticular bleeding, diverticulitis, and fecalith incarceration. These findings are preliminary, and the concept of direct visualization-guided precision endoscopy warrants validation in larger prospective multicenter studies.

## Introduction

Colonic diverticular disease is increasingly recognized as a major gastrointestinal health burden worldwide, particularly in aging populations (1). Although many patients remain asymptomatic, complicated diverticular disease may result in clinically significant manifestations, including diverticular bleeding, diverticulitis, perforation, abscess formation, and fecalith impaction (2–4). Among these, colonic diverticular bleeding represents one of the leading causes of acute lower gastrointestinal hemorrhage (5), whereas diverticulitis and diverticular fecalith incarceration may cause localized inflammation, recurrent abdominal pain, and, in severe cases, abscess formation or intestinal obstruction (6). Despite advancements in endoscopic management, the treatment of complex diverticular disease remains technically challenging because of the narrow diverticular neck, concealed anatomy, and poor intradiverticular visualization (7).

Conventional colonoscopy plays a central role in the diagnosis and management of diverticular disease; however, it frequently encounters substantial limitations in complex cases. During diverticular bleeding, identifying the responsible vessel or stigmata of recent hemorrhage is often difficult because of intermittent bleeding and the presence of multiple diverticula. Similarly, in diverticulitis or fecalith incarceration, conventional endoscopy may fail to adequately visualize the intradiverticular cavity or inflammatory nidus, thereby limiting targeted intervention. Consequently, management frequently relies on empirical antibiotic therapy, repeated imaging, or surgical consultation when conservative treatment fails (5).

Recent technological advances in direct visualization systems have introduced new possibilities for minimally invasive gastrointestinal interventions. The EyeMax direct visualization system (EyeMax, 3 mm; Micro-Tech, Nanjing, China), a digital single-operator cholangioscopy platform, was initially developed for pancreatobiliary diseases and has demonstrated promising utility in difficult endoscopic procedures requiring precise navigation and direct visualization (8). Owing to its slim profile, enhanced maneuverability, irrigation capability, and ability to provide high-resolution intraluminal visualization, the EyeMax system has demonstrated promising utility in several challenging gastrointestinal conditions, including endoscopic retrograde appendicitis therapy (ERAT) (9), appendiceal bleeding (10), colonic diverticular bleeding (11), and so on. In addition, choledochoscopy-based direct visualization techniques have demonstrated value in other technically demanding scenarios, including the diagnosis of biliary metastases from gastric cancer (12). A common feature of these applications is the need to access anatomically restricted spaces and directly visualize otherwise concealed lesions. These experiences suggest that EyeMax may help overcome some of the technical limitations encountered in complex diverticular disease by enabling precise lesion identification and targeted therapy under direct visual guidance. Nevertheless, evidence supporting its use in colonic diverticular pathology remains limited and largely restricted to isolated case reports.

We therefore conducted a proof-of-concept case series to evaluate the feasibility, safety, and expanding therapeutic applications of the EyeMax direct visualization system in complex colonic diverticular disease. Specifically, we investigated its role in diverticular bleeding, diverticulitis, and diverticular fecalith incarceration, while proposing a potential direct visualization-guided therapeutic strategy for difficult diverticular pathology.

## Materials and methods

### Study design and patient selection

This retrospective proof-of-concept case series was conducted at the Department of Gastroenterology, Shandong Provincial Third Hospital, Shandong University. Patients with complex colonic diverticular disease who underwent EyeMax-assisted endoscopic intervention between January 2024 and July 2026 were screened.

**Figure 1:**
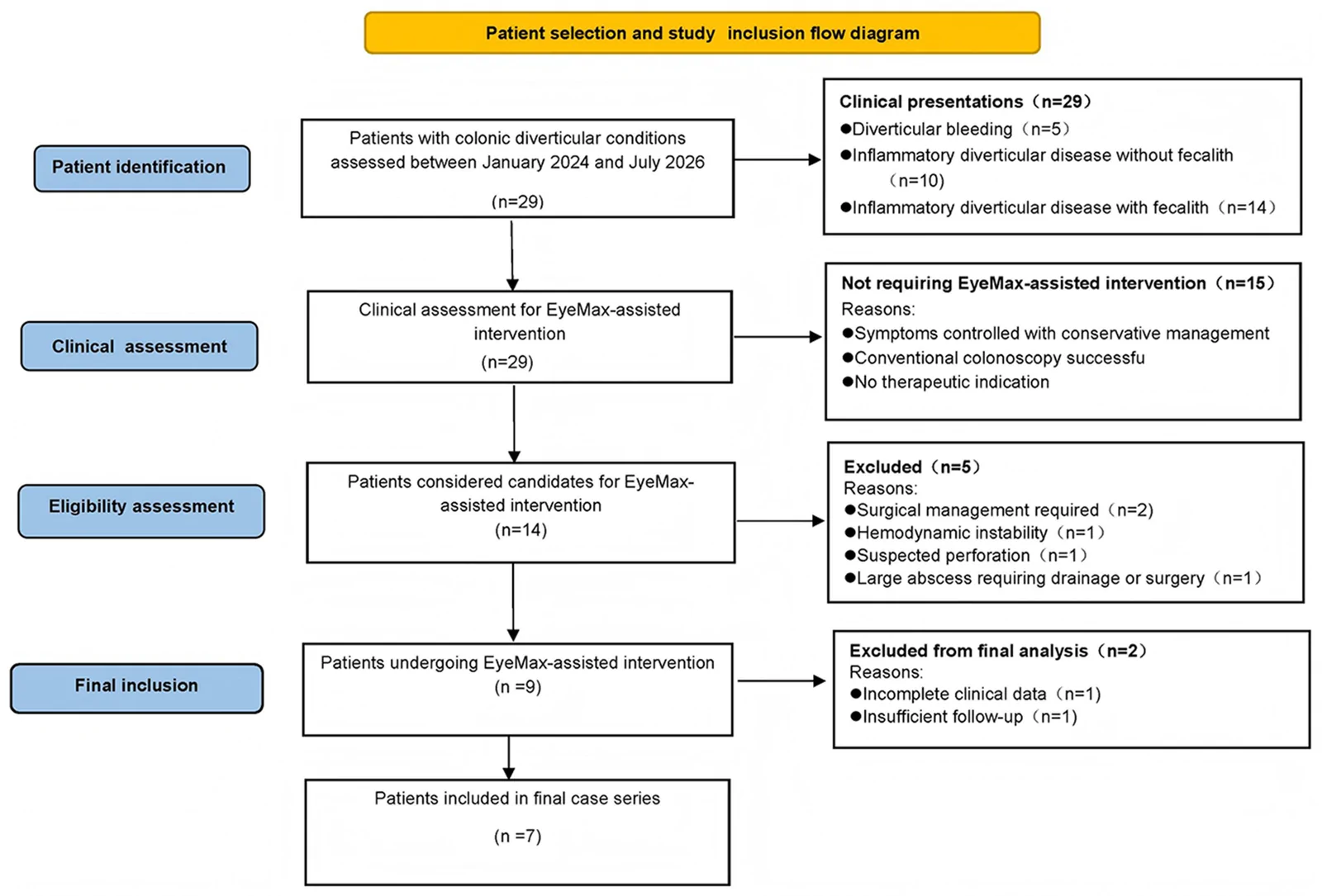
Flow diagram of patient selection for the EyeMax-assisted intervention case series.

In this study, the term complex colonic diverticular disease' was used as an operational definition rather than a formal disease classification. It referred to diverticular conditions in which conventional colonoscopy was insufficient because of limited intradiverticular visualization, difficult access, concealed pathology, or inability to achieve targeted intervention. Eligible patients included those with: (1) colonic diverticular bleeding requiring endoscopic hemostasis; (2) inflammatory diverticular disease requiring direct intradiverticular intervention,including diverticulitis with localized inflammation, purulent cavities, retained fecaliths (Figure 1). Patients included in this case series were independently identified during the study period and were not previously reported. Diverticulitis was diagnosed based on CT findings (e.g., colonic wall thickening, pericolonic fat stranding) and clinical symptoms. Patients with active severe diverticulitis, perforation, generalized peritonitis, large abscess, or hemodynamic instability, suspected malignancy, incomplete clinical data, or insufficient follow-up were excluded. All procedures were performed after clinical assessment, and the decision to perform EyeMax-assisted intervention was made when conventional colonoscopy could not achieve adequate visualization or therapeutic access and when localized endoscopic intervention was considered appropriate.

This study was approved by the institutional ethics committee, and all patients provided informed consent for endoscopic intervention and publication of clinical images.

### EyeMax direct visualization system and endoscopic procedure

All patients underwent standard bowel preparation according to routine colonoscopic protocols. Procedures were performed under conscious sedation or anesthesia according to clinical requirements.

A standard therapeutic colonoscope (CF-HQ290; Olympus Medical Systems, Tokyo, Japan) with a 3.2-mm accessory channel was initially used to identify the target diverticulum. irrigation system (saline irrigation via foot pedal), CO2 insufflation, sedation with propofol (targeted moderate sedation), bowel preparation with polyethylene glycol (4 L) for elective cases and enema for acute bleeding, antibiotic prophylaxis for diverticulitis. When conventional colonoscopy failed to provide adequate intradiverticular visualization or therapeutic access, the EyeMax direct visualization system (9F; Micro-Tech, Nanjing, China) was introduced through the colonoscope accessory channel. The EyeMax system is a miniature direct visualization platform with an outer diameter of 3.0 mm and a 1.2-mm working channel, allowing direct inspection of concealed intradiverticular structures under high-resolution visualization.

**Figure 2:**
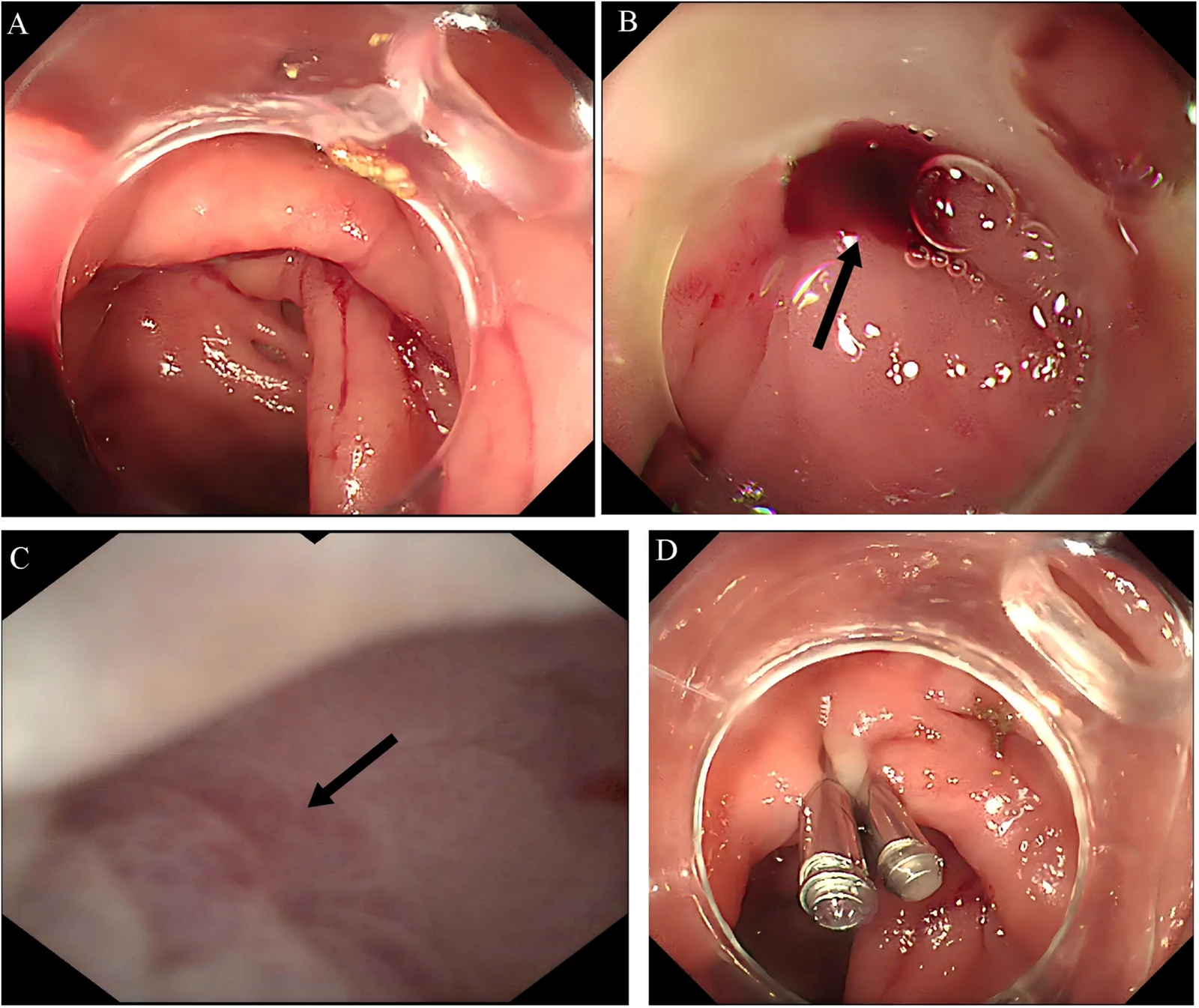
EyeMax-assisted direct visualization-guided hemostasis for diverticular bleeding. **(A)** Conventional colonoscopy demonstrated active lower gastrointestinal bleeding with blood accumulation in the colonic lumen. **(B)** A diverticulum was identified as the suspected bleeding source; however, the diverticular cavity could not be adequately visualized using conventional colonoscopy. The arrow indicates the bleeding diverticulum. **(C)** The EyeMax system was advanced into the diverticular cavity, enabling direct intradiverticular visualization of an exposed culprit vessel (stigmata of recent hemorrhage). The arrow indicates the exposed bleeding vessel. **(D)** Targeted endoscopic hemostasis was performed by complete closure of the diverticular orifice using endoscopic clips after identification of the bleeding source, achieving successful hemostasis.

EyeMax direct visualization system (9 Fr) was introduced through the accessory channel of the colonoscope for direct intradiverticular examination. For diverticular bleeding, direct visualization was used to identify culprit vessels or stigmata of hemorrhage, followed by targeted hemostatic intervention, including multipolar electrocoagulation (coagulation grasper at 30 W) and/or endoscopic clipping (Figure 2).

**Figure 3:**
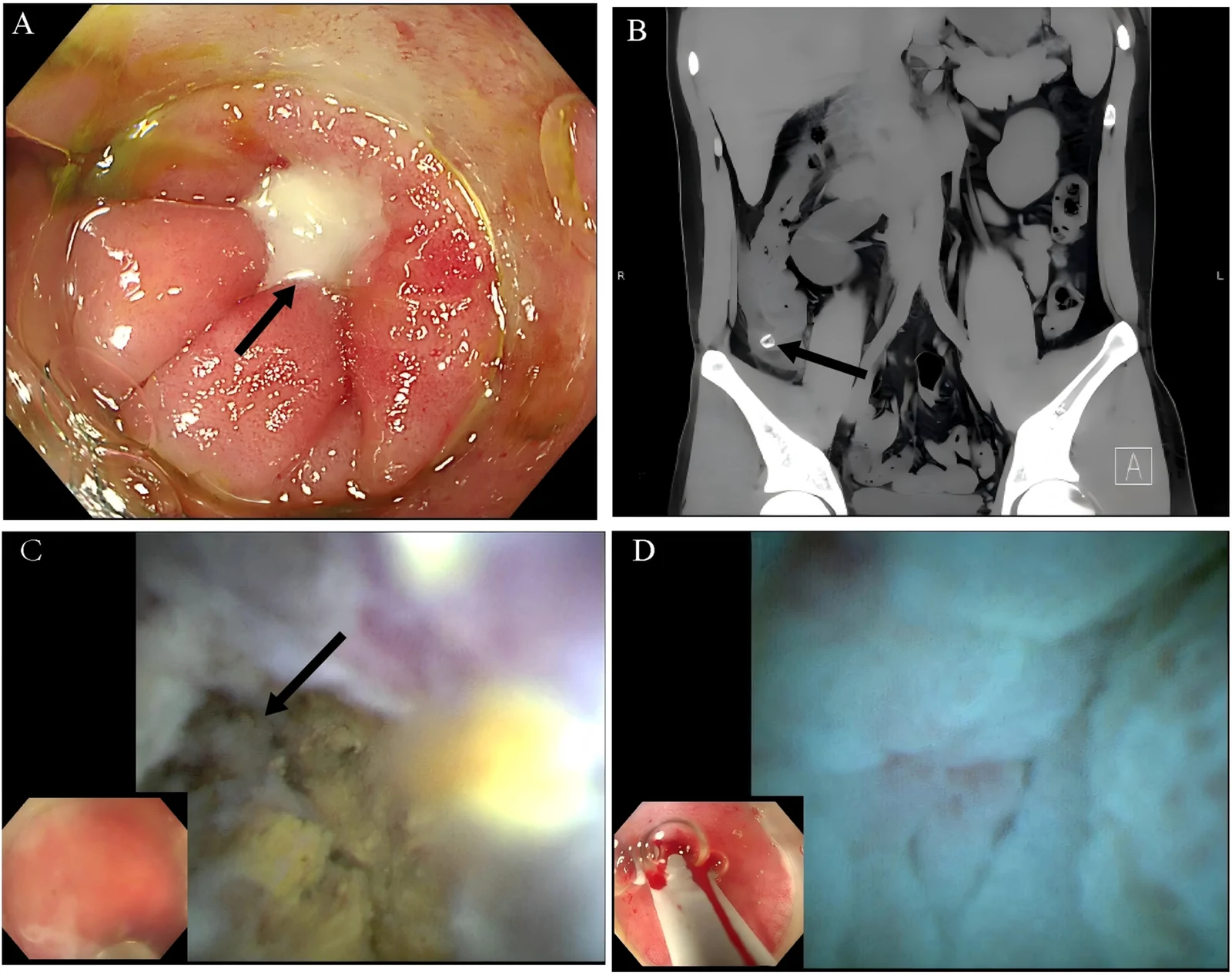
EyeMax-assisted direct visualization-guided intradiverticular intervention for inflammatory diverticular disease. **(A)** Conventional colonoscopy revealed spontaneous purulent discharge emerging from the diverticular orifice, suggesting active inflammatory diverticular disease. The arrow indicates purulent secretion from the diverticular opening. **(B)** Computed tomography demonstrated localized diverticulitis with inflammatory changes surrounding the affected diverticulum. The arrow indicates the inflamed diverticulum and associated intradiverticular fecalith. **(C)** EyeMax-assisted intradiverticular exploration revealed an impacted fecalith, edematous diverticular mucosa, and inflammatory debris within the diverticular cavity. The arrow indicates the retained fecalith. **(D)** Direct visualization-guided irrigation and endoscopic removal of inflammatory materials and fecalith were performed. Repeat EyeMax examination confirmed clearance of the diverticular cavity.

**Figure 4:**
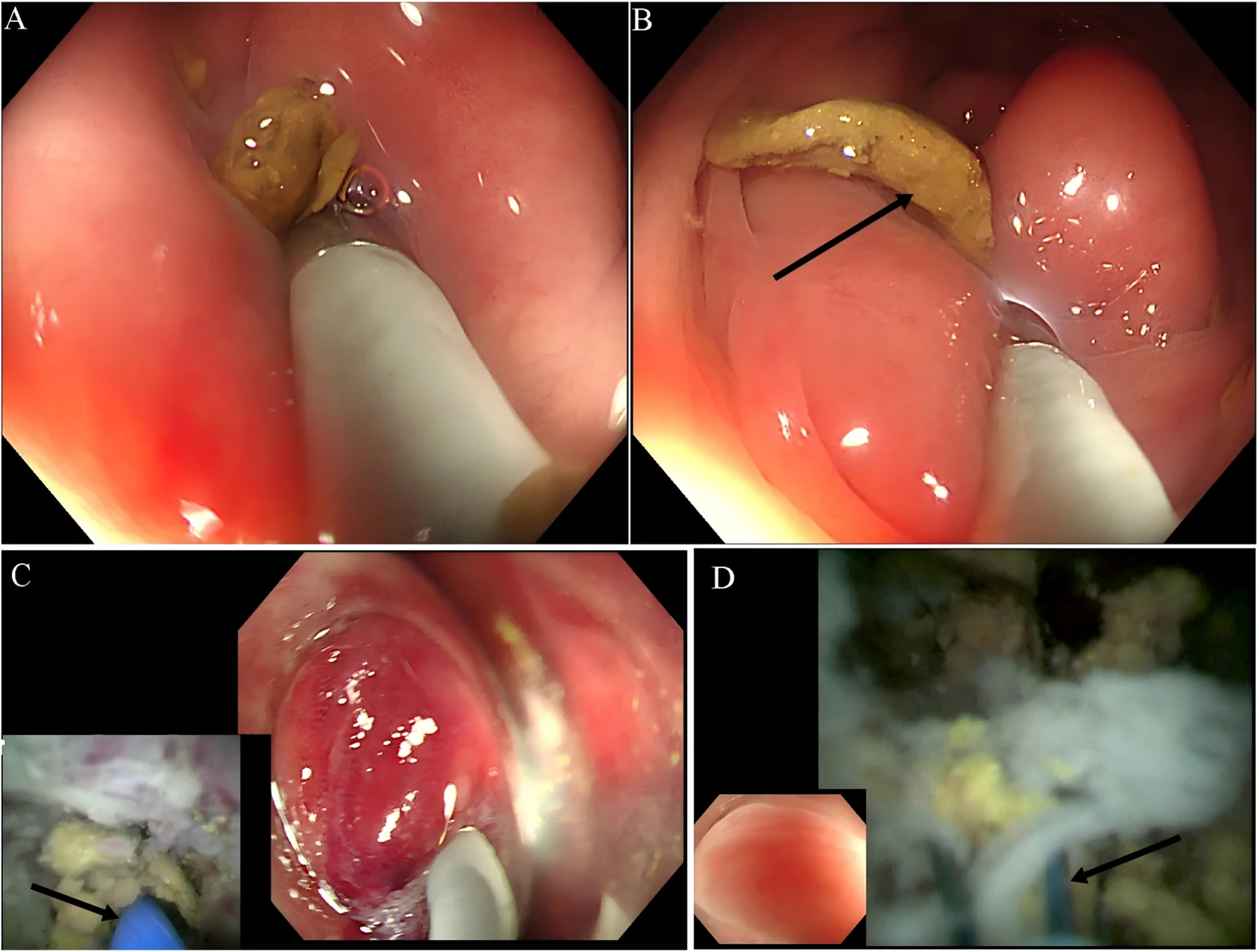
EyeMax-assisted direct visualization-guided management of diverticular fecalith incarceration. **(A)** Continuous irrigation facilitated exposure and mobilization of the impacted fecalith under direct visualization. The arrow indicates the fecalith within the diverticular cavity. **(B)** The fecalith was completely removed under EyeMax guidance. The arrow indicates the extracted fecalith after irrigation, with restoration of a clean diverticular cavity. **(C)** The exposed fecalith was grasped and removed using biopsy forceps. **(D)** Further extraction was performed using a snare under direct visualization. The arrow indicates the snare used for fecalith retrieval.

For inflammatory diverticular disease, EyeMax-assisted intradiverticular exploration was performed to identify purulent material, inflammatory debris, and retained fecaliths. Direct visualization-guided lavage was first used to remove purulent secretions and improve visualization. When retained fecaliths were identified, endoscopic extraction was subsequently performed using biopsy forceps, retrieval baskets, snares, suction-assisted techniques, or a combination thereof, according to the characteristics of the fecalith. Via repeat EyeMax visualization confirmation of clearance (Figures 3, 4).

### Outcome measures

The primary outcome was technical success, defined as successful intradiverticular access with completion of the intended intervention under direct visualization.

Secondary outcomes included:

1. Clinical success, defined as symptom resolution without the need for emergency surgery or additional invasive intervention,for bleeding cases, immediate hemostasis and absence of rebleeding within 30 days; for diverticulitis, resolution of pain, fever within 7 days without need for surgery.
2. Procedure-related adverse events, including perforation, delayed bleeding, infection, or hospitalization related to endoscopic intervention.
3. Recurrence, defined as recurrence of symptoms during follow-up.
4. Need for surgical intervention.

### Data collection and follow-up

Clinical, laboratory, radiologic, endoscopic, procedural, and follow-up data were retrospectively collected from electronic medical records. Collected variables included patient age, sex, diverticular disease subtype, lesion location, imaging findings, procedural details, technical success, clinical outcomes, recurrence, and adverse events.

Patients were followed through outpatient visits or telephone interviews. Follow-up duration ranged from 1 month to 2 years.

### Statistical analysis

Given the exploratory nature and limited sample size of this proof-of-concept case series, statistical analyses were primarily descriptive.

## Results

### Patient characteristics

The study included two patients with diverticular bleeding and five patients with inflammatory diverticular disease, comprising inflammatory diverticular disease without retained fecaliths (*n* = 2), diverticulitis associated with retained fecaliths (*n* = 3). None of the patients included in the current study were reported previously. Lesions were distributed throughout the colon, including the ascending colon, descending colon, and sigmoid colon. Conventional colonoscopy alone was insufficient for accurate localization, visualization, or definitive management in all cases, prompting the use of the EyeMax direct visualization system. Baseline clinical characteristics, laboratory findings, radiological features, procedural details, and follow-up duration are summarized in Table 1.

**Table 1:** Baseline characteristics, intradiverticular findings, procedural details, and clinical outcomes of patients undergoing EyeMax-assisted intervention for complex colonic diverticular disease.

Case	1	2	3	4	5	6	7
Disease subtype	Diverticular bleeding	Diverticular bleeding	Inflammatory diverticular disease without fecalith	Inflammatory diverticular disease without fecalith	Inflammatory diverticular disease with fecalith	Inflammatory diverticular disease with fecalith	Inflammatory diverticular disease with fecalith
Sex	Female	Male	Female	Female	Male	Male	Male
Age	70	55	62	44	24	50	47
Comorbidities and relevant medications	Hypertension and coronary artery disease，oral anti-platelet medication	Coronary artery disease，oral anti-platelet medication	No	No	No	No	No
Location	Descending colon	Sigmoid colon	Ascending colon	Ascending colon	Ascending colon	Ascending colon	Ascending colon
Clinical findings	Hematochezia	Hematochezia	CT localized inflammation	Leukocytosis, CT localized inflammation	Leukocytosis	Persistent abdominal symptoms	Persistent abdominal symptoms
white blood cell count(3.5–9.5 × 10^9/L)	5.9	6.74	5.16	5.7	6.01	10.4	12.58
C-reactive protein（0–3 mg/L）	NR	2.4	2.2	123.2	11.79	185.7	3.25
Hemoglobin (130–175 g/L)	92	89	133	91	147	151	152
EyeMax-assisted intervention	Electrocoagulation + clipping	Clipping	Direct visualization-guided lavage + biopsy forceps and a snare	Direct visualization-guided lavage	Direct visualization-guided lavage	Lavage	Lavage + biopsy forceps/ snare
Previous treatment	No	No	No	No	No	Antibiotic	No
Presence of abscess or perforation	No	No	No	No	No	Abscess	No
Antibiotic use	No	No	Yes	Yes	Yes	Yes	Yes
Technical success	Yes	Yes	Yes	Yes	Yes	Yes	Yes
procedure duration(minutes)	32	28	15	25	16	45	21
Clinical success	Yes	Yes	Yes	Yes	Yes	No	Yes
Duration of hospitalization	8d	7d	5d	6d	4d	11d	3d
Need for surgery	No	No	No	No	No	Yes	No
Follow-up	24 months	12 months	24 months	1 months	2 months	4month	2month
Recurrence	No	No	No	No	No	No	No

### Technical outcomes

EyeMax-assisted intervention achieved technical success in all seven patients (7/7, 100%),procedure duration (mean 26 min, range 15–45 min). In each case, the EyeMax system was successfully advanced into the target diverticular cavity, allowing direct visualization of the underlying pathology and completion of the planned intervention.

No procedure was terminated because of inability to access the diverticular cavity or inadequate visualization.

### Outcomes of diverticular bleeding

Among the two patients with diverticular bleeding, EyeMax-assisted examination enabled direct identification of the bleeding source within the diverticular cavity. In one patient, active bleeding was observed during EyeMax examination, whereas the other patient demonstrated a visible stigma of recent hemorrhage with an exposed vessel. Targeted endoscopic hemostasis was performed using electrocoagulation and/or endoscopic clipping according to the endoscopic findings. Immediate hemostasis was achieved in both patients. During follow-up, neither patient experienced recurrent diverticular bleeding. The follow-up durations of the two bleeding patients were 24 months and 12 months, respectively.

### Outcomes of inflammatory diverticular disease

Among the five patients with inflammatory diverticular disease, EyeMax-assisted intradiverticular exploration demonstrated localized inflammatory changes in all cases, including purulent secretion, inflammatory debris, or retained fecaliths. Three patients had impacted fecaliths within the diverticular cavity. Under direct visualization, fecalith extraction was performed using a combination of endoscopic accessories, including biopsy forceps, retrieval baskets, snares, and suction-assisted techniques according to the size and consistency of the fecalith. Two patients had inflammatory exudate without retained fecaliths. In these patients, direct visualization-guided irrigation was performed to remove purulent material and inflammatory debris, followed by repeat intradiverticular inspection.

Clinical improvement was observed in four of the five patients following EyeMax-assisted intervention. One patient with a history of recurrent diverticulitis presented with an acute exacerbation accompanied by severe localized inflammation. Despite EyeMax-assisted irrigation and removal of inflammatory materials, symptoms persisted. surgery was performed on day 3 due to clinical deterioration, operative findings showed phlegmon without perforation, pathology confirmed diverticulitis, and the endoscopic procedure was not contributory to deterioration. The patient subsequently underwent surgical resection. Detailed clinical course, imaging findings, surgical findings, and pathological results are summarized in Table 1.

### Safety outcomes

No procedure-related perforation, delayed bleeding, procedure-related infection, or mortality was observed during hospitalization or follow-up.

However, given the limited sample size, the absence of observed adverse events does not exclude the possibility of uncommon complications.

### Follow-up outcomes

Follow-up duration ranged from 1 month to 2 years (Table 1).

Follow-up assessment was performed through outpatient evaluation and/or telephone interviews. Recurrence was assessed based on recurrent symptoms, emergency visits, repeat endoscopic intervention, hospitalization, or additional surgical treatment.

During follow-up, no recurrent diverticular bleeding or recurrent inflammatory episodes requiring additional intervention were documented among the six patients who achieved clinical improvement. The patient requiring surgery after failed endoscopic management was considered a clinical failure.

## Discussion

### Key findings

This proof-of-concept case series suggests that the EyeMax direct visualization system appears technically feasible and potentially useful in carefully selected patients with complex colonic diverticular disease. Although complex colonic diverticular disease is not an established diagnostic category, we used this term to describe a clinically challenging spectrum of diverticular conditions characterized by anatomical limitations and the need for direct visualization-guided intervention. Across seven patients with diverticular bleeding, diverticulitis, EyeMax-assisted intervention achieved a technical success rate of 100% with no procedure-related perforation or mortality. More importantly, direct visualization enabled precise identification of concealed pathological targets, including culprit vessels, purulent cavities, inflammatory debris, and impacted fecaliths that could not be reliably visualized using conventional colonoscopy. These findings suggest that the principal advantage of EyeMax lies not simply in its smaller caliber but in its ability to transform endoscopic management from indirect luminal inspection to lesion-directed intervention under continuous visual guidance. Rather than representing three independent disease entities, diverticular bleeding, inflammatory diverticular disease appear to share a common procedural challenge: the pathological process is located within a confined diverticular cavity that is difficult to access using conventional endoscopic techniques. This observation provides the conceptual basis for a unified therapeutic strategy centered on direct visualization of concealed pathological spaces.

Complex colonic diverticular disease remains a significant challenge in gastrointestinal endoscopy because conventional colonoscopy frequently fails to adequately visualize intradiverticular pathology. Conventional colonoscopes are designed primarily for luminal examination rather than exploration of narrow diverticular cavities. Consequently, the narrow diverticular neck, tortuous anatomy, and unstable scope position often limit access to the diverticular lumen and prevent precise identification of culprit lesions, in diverticular bleeding, intermittent hemorrhage and the presence of multiple diverticula may obscure the responsible vessel, resulting in delayed treatment or recurrent bleeding (13–16). Likewise, in diverticulitis or diverticular fecalith incarceration, inflammatory debris, purulent collections, or impacted fecaliths may remain hidden within the diverticular cavity, rendering standard endoscopic therapy difficult or ineffective (17).

### Potential role of EyeMax in diverticular bleeding and inflammatory diverticular disease

In diverticular bleeding, successful endoscopic treatment critically depends on accurate localization of the culprit vessel or stigmata of recent hemorrhage. However, conventional colonoscopy frequently fails to identify bleeding sources because hemorrhage may stop spontaneously or originate from hidden diverticular vessels (18). Previous studies have shown that rebleeding risk remains substantial when definitive hemostasis cannot be achieved (13, 15).

In the present series, EyeMax-assisted exploration allowed direct visualization of culprit vessels within the diverticular neck or lumen, thereby facilitating precise hemostatic intervention. By allowing direct entry into the diverticular cavity, the EyeMax system enabled precise identification of culprit vessels and targeted hemostatic therapy under continuous visualization. This lesion-directed approach has the potential to improve procedural precision, reduce unnecessary intervention, and decrease the likelihood of recurrent hemorrhage.

The most novel finding of this study may be the application of EyeMax-assisted intervention in complicated diverticulitis, particularly through a strategy of direct visualization-guided source control. This strategy extends the concept of source control from surgery to therapeutic endoscopy by enabling direct elimination of the intradiverticular inflammatory nidus.

Current management of uncomplicated diverticulitis generally relies on conservative treatment with antibiotics, while more severe disease may require percutaneous drainage or surgery (19). Increasing evidence suggests that retained fecaliths, inflammatory debris, and localized purulent cavities may serve as persistent inflammatory niduses that perpetuate symptoms and increase recurrence risk (17). In diverticulitis cases in our series, EyeMax-assisted intradiverticular examination revealed purulent material and impacted fecaliths within the diverticular cavity. Under direct visualization, targeted lavage was successfully performed, allowing clearance of purulent contents and local inflammatory debris.

Similarly, diverticular fecalith incarceration represents a technically challenging condition because deeply impacted fecaliths are often inaccessible with conventional colonoscopy. Blind extraction attempts may increase the risk of mucosal injury or perforation.

In our study, instead of considering diverticulitis and fecalith incarceration as two independent disease entities, we regard them as different stages within the same inflammatory spectrum of diverticular disease. EyeMax-assisted intervention enabled direct visualization of the impacted fecalith and facilitated targeted extraction using biopsy forceps, basket retrieval, and suction-assisted techniques. This direct visualization strategy improved procedural precision and may reduce unnecessary manipulation of surrounding tissue.

### Direct visualization-guided precision endoscopy: A unified therapeutic concept

An important conceptual contribution of this study is the proposal that EyeMax may function as a unified therapeutic platform across the spectrum of complex diverticular disease, which direct visualization becomes the central determinant of procedural precision across anatomically concealed gastrointestinal diseases.

Our experience suggests that direct visualization-assisted endoscopy may address these common limitations through four principal mechanisms:

1. Enhanced access to difficult diverticula through a slim-profile platform;
2. Direct visualization of culprit vessels, purulent cavities, or impacted fecaliths;
3. Precision intervention using targeted hemostasis, lavage, or extraction;
4. Potential safety improvement by reducing blind manipulation.

Based on these findings, we propose a conceptual framework in which EyeMax-assisted intervention may represent an emerging minimally invasive strategy for difficult diverticular pathology, particularly in patients who fail conventional endoscopic management or are poor surgical candidates. Rather than considering EyeMax solely as another ultra-slim endoscope, it may be more appropriately regarded as a direct visualization platform capable of expanding precision endoscopy into anatomically concealed gastrointestinal diseases.

### Limitations

Several limitations should be acknowledged. First, this was a retrospective single-center study with a limited sample size, introducing the possibility of selection bias. Second, the absence of a control group precludes direct comparison with conventional colonoscopy or alternative endoscopic techniques. Third, the study included different manifestations of diverticular disease, resulting in clinical heterogeneity. Nevertheless, this heterogeneity also supports our central hypothesis that these seemingly distinct conditions share a common procedural challenge that may be addressed using a unified direct visualization strategy. Finally, appears technically feasible and potentially useful in carefully selected patients. We acknowledged that the absence of adverse events in seven patients does not establish safety and that uncommon serious events cannot be excluded. Longer follow-up and multicenter prospective studies are required to evaluate long-term durability, learning curves, cost-effectiveness, and generalizability.

## Conclusion

The EyeMax direct visualization system appears to be a feasible endoscopic platform for the management of complex colonic diverticular disease in this preliminary assessment. While our findings suggest potential for precision hemostasis and expanding applications such as direct visualization in inflammatory diverticular disease and precise intervention for diverticular fecalith incarceration, these efficacy and safety observations remain preliminary. The concept of Direct Visualization-Guided Precision Endoscopy warrants validation in larger prospective multicenter studies.

## Data Availability

The original contributions presented in the study are included in the article/Supplementary Material, further inquiries can be directed to the corresponding author.
